# Hormone replacement therapy and cardiovascular risk in postmenopausal women

**DOI:** 10.1093/ehjopen/oeag054

**Published:** 2026-03-28

**Authors:** Isabella Blackburn, Vijay Kunadian

**Affiliations:** Translational and Clinical Research Institute, Faculty of Medical Sciences, Newcastle University, 4th Floor William Leech Building, Newcastle upon Tyne NE2 4HH, UK; Translational and Clinical Research Institute, Faculty of Medical Sciences, Newcastle University, 4th Floor William Leech Building, Newcastle upon Tyne NE2 4HH, UK; Cardiothoracic Centre, Freeman Hospital, Newcastle upon Tyne Hospitals NHS Foundations Trust, Newcastle upon Tyne NE7 7DN, UK

**Keywords:** Cardiovascular disease, Coronary heart disease, Menopause hormone replacement therapy, Postmenopausal women, Stroke, Venous thromboembolism

## Abstract

Menopause hormone replacement therapy (HRT) remains the main strategy for managing menopausal symptoms and preventing osteoporosis in postmenopausal women. However, its cardiovascular effects are complex and influenced by multiple factors. Early initiation of HRT within 10 years of menopause onset consistently demonstrates cardiovascular benefits, whereas delayed initiation may increase risks such as stroke and venous thromboembolism. Transdermal and bioidentical hormones generally show a safer cardiovascular profile compared to oral synthetic preparations. Current guidelines advocate for individualized therapy considering patient preferences and risk stratification. However, significant knowledge gaps remain regarding long-term safety, diverse populations, and optimized risk assessment tools. The development of a menopause-specific cardiovascular risk calculator could enhance patient-centred care and guide shared decision-making. This review synthesizes current evidence from major randomized trials, observational studies, and meta-analyses, highlighting the critical role of timing, hormone formulation, administration route, and baseline cardiovascular risk in determining HRT’s cardiovascular outcomes. It also underscores the importance of precision medicine in optimizing cardiovascular and overall health outcomes for postmenopausal women using HRT.

## Graphical Abstract

**Graphical Abstract:**
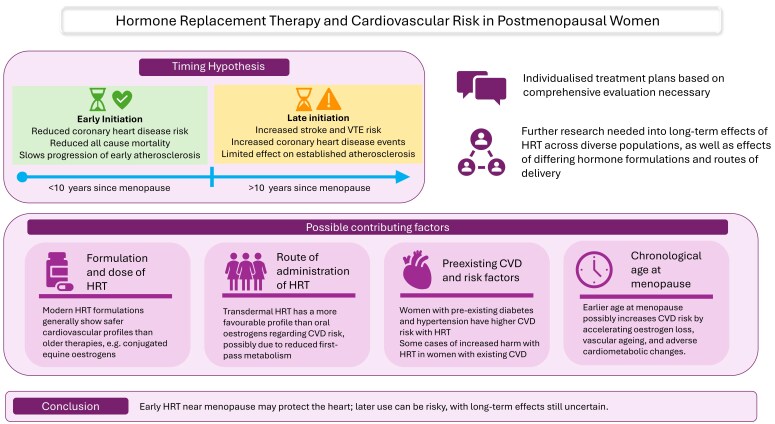

## Introduction

Cardiovascular disease (CVD) remains the leading cause of morbidity and mortality among women in the UK, surpassing deaths from all forms of cancers combined.^1,2^ Over 3.6 million women are living with heart and circulatory disease in the UK.^3^ The risk of CVD increases significantly during the menopause transition, in part due to the decline in endogenous oestrogen, which plays a crucial role in maintaining vascular tone, lipid metabolism, endothelial function, and modulating inflammatory pathways.^4,5^ Epidemiological data, including large cohort studies and meta-analyses, have consistently shown that both early menopause and postmenopausal status are independent risk factors for CVD, separate from the effects of chronological ageing.^6^

Hormone replacement therapy (HRT), typically involving oestrogen alone or in combination with a progestogen, is widely prescribed to alleviate vasomotor symptoms of menopause and to prevent postmenopausal bone loss.^7^ However, its role in cardiovascular health has been the subject of extensive debate. This controversy gained significant public attention following the publication of the Women’s Health Initiative (WHI) trial in the early 2000s.^8^ This landmark randomized trial reported increased risks of breast cancer, stroke, and coronary heart disease (CHD) in older women taking combined oestrogen–progestogen therapy.^8,9^ Similarly, the UK-based Million Women Study, published in 2003, also found an increased incidence of breast cancer among HRT users, particularly those with combined regimens.^10^ These findings, widely publicized in the media, led to a dramatic decline in HRT use and contributed to fear and uncertainty among both patients and clinicians.^9,11^ These findings, largely derived from older populations, may have led younger symptomatic women to avoid HRT despite potential therapeutic benefit.^11^

Subsequent reanalyses and newer observational studies have refined understanding of HRT’s cardiovascular effects. Given this evolving evidence, this review aims to synthesize current knowledge regarding HRT and cardiovascular risk in postmenopausal women. It explores the mechanistic basis of hormone action, evaluates evidence from major clinical trials and observational studies, and highlights how individual risk factors such as age at initiation, baseline cardiovascular status, and HRT formulation influence outcomes. Guidelines from major societies, including the British Menopause Society, now emphasize individualized risk assessment and shared decision-making between the multi-disciplinary team.^7^ The review also discusses the implications for clinical decision-making considering updated guidelines that prioritize personalized, evidence-based care.

## Mechanistic basis of hormone replacement therapy and cardiovascular risk

The decline in endogenous oestrogen production following menopause is a key factor contributing to the increased CVD risk observed in postmenopausal women. Notably, the incidence of CHD in women lags approximately 10 years behind that in men, an effect thought to be largely due to the cardioprotective influence of oestrogen during the reproductive years.^12^ Oestrogen modulates numerous vascular, metabolic, and inflammatory pathways that collectively support cardiovascular health in premenopausal women.^5,13^ While HRT was initially believed to restore these benefits, subsequent research has demonstrated that the cardiovascular effects of exogenous oestrogen are complex, time-dependent, and highly influenced by formulation, dose, and route of administration.^13^Oestrogen promotes angiogenesis and vasodilation, improving vascular function and blood flow.^14^ This is partly mediated by the activation of endothelial nitric oxide (NO) synthase, which increases NO production, a potent vasodilator.^15^ Oestrogen also reduces cardiac fibrosis and oxidative stress, contributing to improved myocardial health and reduced vascular injury.^14^ It may raise HDL cholesterol levels, which is associated with a lower risk of CVD.^14^ Clinical studies have shown that oestrogen improves endothelial function, as evidenced by enhanced flow-mediated dilation (FMD), a validated marker of vascular health.^15^

Experimental studies also suggest that later menopause may preserve endothelial mitochondrial function and reduce oxidative stress, which may partially explain the lower CVD risk observed in these women.^16^ Serum from late-onset postmenopausal women induces less mitochondrial reactive oxygen species (mitoROS) related bioactivity in endothelial cells, which is linked to a more favourable circulating lipid profile, especially lower levels of triglyceride-derived metabolites such as TG(16:0).^16^ Normalizing TG(16:0) levels abolishes differences in mitoROS bioactivity, indicating its causal role.^16^ These findings suggest that later menopause age reduces CVD risk by preserving endothelial function, thereby improving vascular function and blood flow.^16^

Additionally, oestrogen appears to inhibit the initiation of fatty streaks in arterial walls, an early step in atherosclerosis development, although it may not prevent progression of advanced lesions once established.^17^ Some randomized controlled trials (RCTs) have demonstrated that when initiated early in the postmenopausal period, oestrogen therapy may slow the progression of coronary artery atherosclerosis.^15,18^

Epidemiological data suggest that a longer duration of endogenous oestrogen exposure, such as with later menopause, is associated with a reduced risk of CVD, suggesting a protective role of natural oestrogen throughout a woman’s life.^13^ Large pooled analyses show that women who experience early menopause (before age 45 years) or premature menopause (before age 40) have a significantly increased risk of cardiovascular events compared to those who undergo menopause at the typical age of 50–51 years.^6,19^ This increased risk persists even after adjusting for confounding factors such as smoking, body mass index (BMI), and education level.^6^ This risk is particularly pronounced before age 60 and attenuates with advancing age, indicating that menopause timing has a stronger impact on CVD risk earlier in postmenopausal life.^6^

**Figure 1:**
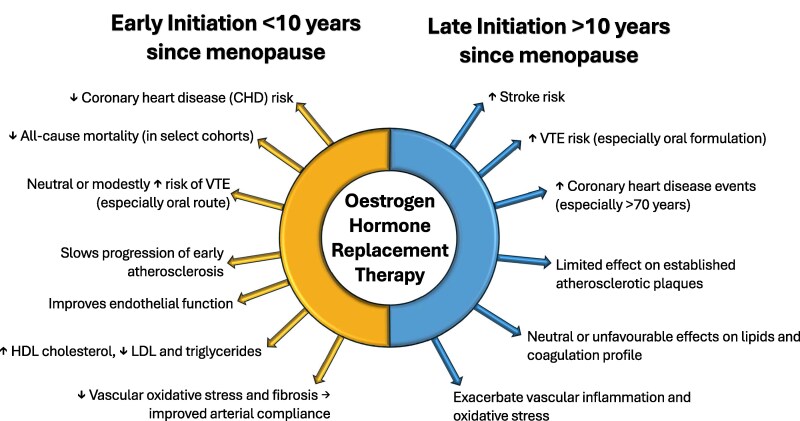
Timing hypothesis of hormone replacement therapy (HRT) and cardiovascular risk. Early initiation of HRT (within 10 years of menopause or before age 60) is associated with a more favourable CVD profile, while later initiation is linked to higher risks. Adapted from major society guidelines. CVD, cardiovascular disease; HDL, high-density lipoprotein; HRT, hormone replacement therapy; LDL, low-density lipoprotein; VTE, venous thromboembolism.

The ‘timing hypothesis’ proposes that the cardiovascular effects of HRT are age- and time-sensitive.^15,18^ Initiating HRT close to the onset of menopause may reduce CHD and all-cause mortality, whereas delayed initiation, particularly beyond 10 years post-menopause, may not confer these benefits and could even increase cardiovascular risks, as illustrated in *Figure 1*.^15,18^ Despite these potential benefits, oestrogen therapy, especially when combined with a progestogen, has been associated with increased risk of stroke, venous thromboembolism (VTE), and ischaemic heart disease (IHD) in several large-scale randomized trials.^14,20^

## Clinical evidence on cardiovascular outcomes

### Randomized controlled trials

The WHI landmark RCT evaluated the effects of combined oral conjugated equine oestrogen (CEE) and medroxyprogesterone acetate (MPA) in postmenopausal women aged 50–79 years.^8^ This arm of the study was terminated prematurely in 2002 due to findings of increased risks of CHD, stroke, and VTE in the hormone therapy group compared with placebo.^11^ These results challenged earlier observational studies suggesting cardioprotective effects of HRT and raised concerns, particularly about initiating HRT in older women.^8^ The WHI also found that late initiation of combined therapy increased risk of CHD, with persistent stroke risk from the second year, in predominantly healthy postmenopausal women.^8^ Importantly, the trial tested only one formulation and delivery route of HRT (oral CEE + MPA), limiting generalizability to contemporary regimens such as transdermal oestradiol or micronized progesterone.^8^

The Heart and Oestrogen/Progestin Replacement Study (HERS), published in 1998, focussed on secondary prevention in postmenopausal women with existing CHD.^21^ The study found no reduction in CHD outcomes with CEE + MPA and reported an early increase in coronary events and myocardial infarction during the first year of therapy, alongside increased VTE risk but with no significant effect on stroke.^21^ These findings contributed to the growing understanding that HRT may not be appropriate for women with established heart disease.^14^

The Early vs. Late Intervention Trial with Oestradiol (ELITE) trial specifically tested the timing hypothesis by comparing the effects of oral oestradiol on atherosclerosis progression in women initiating therapy either <6 years or ≥10 years after menopause.^17^ Oestradiol significantly slowed carotid artery intima-media thickness progression in the early intervention group but had no effect in those further from menopause, demonstrating that oestrogen therapy initiated soon after menopause may slow atherosclerosis progression in recently postmenopausal women without pre-existing disease.^17^

The Kronos Early Oestrogen Prevention Study (KEEPS), published in 2013, investigated cardiovascular outcomes in recently menopausal women using oral CEE or transdermal 17β-oestradiol combined with cyclic progesterone.^22^ Although smaller in scale, KEEPS supported the timing hypothesis, demonstrating favourable vascular profiles and minimal adverse events when therapy was started early after menopause. The trial also reported no significant differences in carotid intima-media thickness progression or coronary artery calcification among treatment groups, indicating no adverse cardiovascular outcomes during the 2005–08 study period.^22^ However, many early trials enrolled women substantially older than the typical age of HRT initiation in clinical practice, which may limit the applicability of their findings to younger symptomatic women.^8,9^

### Observational studies and registries

The Danish Osteoporosis Prevention Study (DOPS) was a prospective, investigator-initiated, multicentre study that investigated the effects of HRT in recently postmenopausal women aged 45–58 years.^18^ The DOPS demonstrated that initiating HRT shortly after menopause was associated with a reduction in the composite endpoint of death, heart failure, and myocardial infarction. This cardiovascular benefit persisted not only during the 10-year intervention period but also throughout an additional 6 years of follow-up after treatment cessation.^18^ Importantly, no increased risk of cancer, stroke, or VTE was observed in the treatment group. These findings support to the timing hypothesis, emphasizing the importance of early HRT initiation in relation to menopause onset for achieving cardiovascular benefits.^18^

A more recent Swedish nationwide register-based study examined the impact of contemporary HRT on CVD risk in women aged 50–58 years, representing the typical menopausal transition period.^14^ This large-scale register-based cohort study analysed national prescribing and cardiovascular outcomes from July 2007 to December 2018, including 919 614 women, of whom 77 512 initiated HRT. One of the key strengths of this study was its inclusion of a wide range of HRT formulations, such as oral combined continuous and sequential regimens, oral unopposed oestrogen, oral oestrogen with local progestogen, tibolone, transdermal combined, and transdermal unopposed oestrogen.^14^

Oral continuous combined HRT was linked to a modest increase in the risk of IHD, whereas transdermal oestrogen was not associated with a significant increase in CVD risk.^14^ Over the course of the study, the overall incidence of CVD, especially IHD, declined by more than 50%, coinciding with a shift in HRT prescribing regimens towards transdermal formulations as well as the use of oral oestrogen in combination with a levonorgestrel-releasing intrauterine system.^14^ Moreover, the findings suggest that modern HRT formulations may carry a lower stroke risk compared with older therapies, such as CEE.^14^

### Meta-analyses and systematic reviews

A comprehensive systematic review and meta-analysis published in 2024 evaluated the cardiovascular effects of HRT across 33 RCTs involving 44 639 postmenopausal women.^15^ The analysis revealed that HRT did not significantly reduce all-cause mortality (RR = 0.96) or major cardiovascular events (RR = 0.97). However, HRT was associated with an increased risk of both stroke and VTE, each with a relative risk of 1.86.^15^ Hormone replacement therapy significantly improved endothelial function, as measured by FMD, with a standardized mean difference of 1.46. In contrast, no meaningful improvement was noted in nitroglycerine-mediated dilation, suggesting a limited impact on endothelium-independent vascular function.^15^

Timing of initiation emerged once again as a critical factor influencing outcomes. Women who began HRT within 10 years of menopause experienced lower rates of all-cause mortality and cardiovascular events, alongside greater improvements in FMD, compared with those initiating therapy later.^15^ Interestingly, no significant differences were observed in cardiovascular outcomes between mono-oestrogen therapy and combined oestrogen–progestogen therapy.^15^ The review followed established PRISMA and GRADE guidelines and incorporated trial sequential analysis to evaluate the robustness of the evidence. Limitations included heterogeneity in FMD and nitroglycerine-mediated dilation outcomes, as well as incomplete data regarding hormone dose, administration route, and treatment duration.^15^

A separate meta-analysis explored the combined effects of physical exercise and HRT on cardiovascular and metabolic health outcomes.^23^ This review included seven RCTs involving 386 women aged 48–58 years. The findings indicated that the combination of aerobic training and oral HRT led to a greater reduction in systolic blood pressure compared to aerobic training alone.^23^ However, the combined intervention attenuated improvements in diastolic blood pressure and peak oxygen consumption (VO_2_ peak) promoted by exercise, suggesting a potential counteractive effect of oral HRT on certain fitness outcomes. Notably, transdermal oestrogen appeared to yield more favourable effects on blood pressure compared to oral formulations.^23^ Despite these insights, the overall quality of evidence in this review was rated as low to very low, largely due to small sample sizes and methodological limitations within the included studies.^23^ As such, while the interaction between exercise and HRT remains a promising area of research, further high-quality studies are needed to clarify their combined cardiovascular effects. The key findings from the major clinical trials and observational studies discussed are summarized in *Table 1*.

**Table 1:** Summary of key trials with population, intervention, outcome, and findings

Study	Population	Intervention	Outcomes measured	Key findings
WHI (Women’s Health Initiative)	16 608 postmenopausal women aged 50–79	CEE ± MPA	CVD events, stroke, VTE, breast cancer, mortality	No CVD benefit overall Increased risk of coronary events, stroke, and VTE especially in late initiators
HERS (Heart and Oestrogen/Progestin Replacement Study)	2763 postmenopausal women with established CHD aged <80 years	CEE + MPA	Secondary prevention of coronary events	No reduction in coronary events Some early increased risk No overall CVD benefit
KEEPS (Kronos Early Oestrogen Prevention Study)	727 recently postmenopausal women aged 42–58 years	Oral CEE or transdermal oestradiol + progesterone	Subclinical atherosclerosis, vascular function	Early initiation showed improved vascular function No major adverse coronary events
ELITE (Early vs. Late Intervention Trial with Oestradiol)	643 postmenopausal women, stratified by time since menopause (<6 vs. >10 years)	Oral oestradiol ± progesterone	Carotid intima-media thickness progression	Early initiation (<6 years) slowed atherosclerosis progression Late initiation showed no benefit
DOPS (Danish Osteoporosis Prevention Study)	1006 recently postmenopausal women aged 45–58	Oral oestradiol ± norethisterone acetate	Composite of death, heart failure, myocardial infarction	HRT initiated soon after menopause reduced CVD events without increased cancer or VTE risk
Swedish Register Study (emulated target trial)	77 512 HRT users among 919 614 total women aged 50–58 years	Various HRT formulations (oral/transdermal, combinations)	CVD incidence, IHD, stroke, VTE	Transdermal oestrogen had no increased CVD risk Oral combined HRT slightly increased IHD risk Overall CVD declined over time
Gu et al., 2024 Meta-analysis	44 639 postmenopausal women from 33 RCTs	Various HRT regimens	All-cause mortality, CVD, stroke, VTE, endothelial function (FMD)	HRT did not reduce mortality or CVD events overall Increased stroke and VTE risk Benefits seen with early initiation
Sanchez-Delgado et al., 2023 Meta-analysis	386 women from seven RCTs	Combined aerobic exercise and oral HRT	Blood pressure, VO2 peak, physical fitness	Combined HRT and exercise reduced systolic BP more than exercise alone but attenuated improvements in diastolic BP and VO2 peak

BP, blood pressure; CEE, conjugated equine oestrogens; CHD, coronary heart disease; CVD, cardiovascular disease; FMD, flow-mediated dilation; HRT, hormone replacement therapy; IHD, ischaemic heart disease; MPA, medroxyprogesterone acetate; RCT, randomized control trial; VTE, venous thromboembolism.

## Risk stratification and modifying factors

### Type and route of hormone therapy

The formulation and delivery route of HRT significantly influence its cardiovascular effects. Bioidentical hormones, such as 17β-oestradiol and micronized progesterone, are structurally identical to endogenous human hormones and are believed to have a more favourable cardiovascular risk profile compared to synthetic alternatives.^20^ In particular, natural progesterone appears to preserve the vascular benefits of oestrogen, whereas synthetic progestins, notably MPA, may blunt these benefits and have been associated with increased cardiovascular risks.^18,20^

Findings from landmark trials such as the WHI and the HERS, which primarily used CEE combined with MPA, showed no cardiovascular benefit and even increased risks of coronary events, stroke, and VTE, particularly when HRT was initiated later in life.^8,20,21^ Oral synthetic oestrogens have a stronger impact on coagulation factors, increasing the risk of VTE compared to transdermal bioidentical oestrogens.^20,24^

The route of administration also influences cardiovascular outcomes. Oral oestrogens, especially CEE, undergo hepatic first-pass metabolism, stimulating coagulation factor production and the renin-angiotensin-aldosterone system, which may raise diastolic blood pressure and contribute to a pro-thrombotic state.^23^ In contrast, transdermal oestrogen bypasses hepatic metabolism, minimizing these adverse effects. Studies commonly show that transdermal HRT has a neutral or favourable impact on blood pressure regulation and VTE risk.^15,24^ Additionally, transdermal therapy has neutral or favourable effects on triglyceride levels, whereas oral HRT more strongly modifies lipid metabolism, typically reducing LDL cholesterol but increasing triglycerides.^24^ Despite these biochemical differences, overall lipid profile improvements are not significantly different between the two routes.^24^

### Baseline cardiovascular risk

Baseline cardiometabolic status is a major determinant of HRT risk–benefit balance. Obesity, for example, is associated with chronic inflammation, dyslipidaemia, and insulin resistance, all of which may exacerbate cardiovascular risk during HRT use. Interestingly, women with higher BMI tend to have a lower relative risk of breast cancer with HRT, but possibly also face elevated cardiovascular risks due to pre-existing metabolic dysfunction.^10,23^

Menopause is commonly accompanied by metabolic changes, including increased insulin resistance and impaired glucose metabolism. Oral oestrogens have been shown to improve insulin sensitivity and glycaemic control, though the evidence remains limited and somewhat inconclusive.^15,24^ Nonetheless, women with pre-existing diabetes or hypertension are at higher risk for adverse cardiovascular events during HRT, given their pro-atherogenic and pro-thrombotic profiles.^21^

Women with established CVD require additional caution. Randomized controlled trials such as WHI and HERS demonstrated no cardiovascular benefit, and in some cases, increased harm, in older women or those with pre-existing CVD, underscoring the importance of careful risk stratification.^20,21^ A retrospective matched-cohort study of female heart transplant recipients found that post-transplant HRT did not increase rejection, major cardiac events, or mortality; however, further research is needed to accurately establish these findings, given the small sample size of available studies.^25^ Conversely, early HRT initiation in metabolically healthy women may improve lipid profiles and surrogate markers of vascular health, such as carotid intima-media thickness and FMD.^15,18^

### Current guidelines and clinical implications

The cardiovascular effects of HRT are not uniform across all postmenopausal women. Personalized HRT decision-making based on these factors is now central to clinical guidelines from major societies.^7,26^ Contemporary HRT guidelines from major professional bodies including the National Institute for Health and Care Excellence, the British Menopause Society (BMS), and the North American Menopause Society share foundational principles but differ slightly in emphasis, scope, and regional focus.^7,26,27^ Differences between society guidelines regarding HRT use, risk assessment, and clinical decision-making are summarized in *Table 2*. All three agree that HRT is the most effective treatment, particularly for vasomotor symptoms and genitourinary syndrome of menopause. Hormone replacement therapy is also recommended for prevention and treatment of osteoporosis, especially in women under 60 years of age or those with premature ovarian insufficiency.^7,26,27^ A unifying feature of all guidelines is the emphasis on individualized care. Treatment decisions should be based on a comprehensive evaluation of the patient’s symptom profile, cardiovascular and breast cancer risk, personal and family history as well as preferences. Ongoing reassessment is advised to ensure that the benefits continue to outweigh the risks as a woman ages.^7,26,27^

**Table 2:** Comparison chart summarizing key aspects of hormone replacement therapy guidance from National Institute for Health and Care Excellence, British Menopause Society, and the North American Menopause Society

Aspect	NICE (UK)	BMS (UK)	NAMS (USA)
Primary use	Relief of vasomotor and urogenital symptoms; osteoporosis prevention/treatment	Vasomotor symptoms, GSM, osteoporosis; supports broader quality-of-life considerations	Vasomotor symptoms, GSM, osteoporosis prevention
Initiation timing	No strict time limit: earlier initiation preferred but not mandatory	Advocates initiation near menopause; supports long-term use if benefits > risks	Recommends starting <60 years old or within 10 years of menopause onset
Duration of use	No arbitrary limit: regular individualized reassessment recommended	Supports long-term use when clinically indicated and reassessed periodically	Periodic evaluation advised; benefits clearer with early initiation
Cardiovascular risk consideration	CVD risk assessed individually; transdermal preferred in higher-risk patients	Emphasizes timing hypothesis; lower CVD risk when started early and transdermal use preferred	Strong support for timing hypothesis; early use may reduce CHD and mortality
Route preference	No universal preference; transdermal may have lower VTE and stroke risk	Transdermal and micronized progesterone preferred in many cases	Transdermal oestrogen and bioidentical progesterone favoured for better CVD profile
Cancer risk (e.g. breast)	HRT may slightly increase risk; individual risk factors considered	Risk is low in younger women and depends on type, duration, and timing	Risk increases with combined HRT over time; lower with oestrogen-only or transdermal preparations
Shared decision-making	Strongly emphasized; personalized risk–benefit discussion essential	Central to guideline; considers lifestyle, quality of life, symptom burden	Strongly emphasized; encourages collaborative approach
Review frequency	Annual or more often if clinical changes occur	Regular reassessment advised; no fixed schedule	Periodic re-evaluation, particularly if extending therapy beyond 5–10 years

CHD, coronary heart disease; CVD, cardiovascular disease; GSM, genitourinary symptoms of menopause; HRT, hormone replacement therapy; VTE, venous thromboembolism.

North American Menopause Society endorses the ‘timing hypothesis’ and recommends initiation of HRT in women under age 60 or within 10 years of menopause onset, advising periodic re-evaluation for longer durations.^26^ National Institute for Health and Care Excellence has no fixed arbitrary limits on duration but recommends regular review,^27^ and the BMS supports long-term use when there is clear clinical indication, noting that arbitrary limits on duration are not evidence-based.^7^

For clinicians, the evolving guidance underscores the importance of nuanced, evidence-based conversations about HRT with menopausal patients. The decision to initiate HRT should be based not on age alone, but on an assessment of individual risk factors, symptom burden, and therapeutic goals.^27^ Ultimately, modern guidance emphasizes that HRT can be safely used in many women, and the cardiovascular concerns, while valid, should not lead to blanket avoidance in otherwise eligible patients.^28^

### Knowledge gaps and research directions

While earlier natural menopause is linked to increased risk of CVD, the precise biological mechanisms underlying the association remain incompletely understood.^6^ Although the protective role of endogenous oestrogens on vascular function and inflammation is recognized, the pathways by which early oestrogen loss leads to vascular damage and how genetic and environmental factors interact with this process require further elucidation.^5^ Additionally, distinguishing cardiovascular risk changes attributable to the menopausal transition from those due to chronological ageing remains challenging. Some studies suggest that ageing itself or pre-existing cardiovascular risk factors may play a more dominant role than menopausal status, but consensus is lacking.^19,28^

The impact of HRT formulation, dose, and route of administration on cardiovascular outcomes also remains an important area for investigation. Different types of HRT and routes of delivery have varying effects on cardiovascular risk factors. Transdermal oestrogens may pose a lower risk of VTE and stroke compared to oral preparations, but large multicentre randomized trials directly comparing these variables are limited.

The long-term cardiovascular consequences after stopping HRT, especially when initiated early post-menopause, are not well characterized. Some studies report no increased risk after discontinuation, but data is limited.^18^ Another critical gap in understanding is the long-term cardiovascular safety of different HRT formulations across diverse populations. While observational and randomized data support the safety of early HRT initiation in healthy women, there remains limited high-quality evidence on the comparative effects of various progestogens, particularly bioidentical vs. synthetic types.^14,20,24^

Furthermore, most major trials underrepresent diverse racial and ethnic groups, including the WHI,^11^ restricting the generalizability of findings. Additionally, data on women experiencing premature ovarian insufficiency or early menopause, who often require longer durations of hormone therapy, remain scarce. These groups may have distinct cardiovascular risk profiles that are inadequately addressed by current guidelines.^27^

Despite widespread use of HRT to manage menopausal symptoms, there remains a significant gap in the ability to accurately assess cardiovascular risk in women considering HRT.^20^ Research shows that HRT is not uniformly beneficial or harmful for CVD risk, and the cardiovascular effects of HRT vary significantly between individuals.^14,18^ Given these complexities, a dedicated CV risk calculator tailored for women considering or using HRT could help clinicians weigh the benefits and risks more precisely. Currently available CV risk calculators such as QRISK3 and the ASCVD Pooled Cohort Equations are designed for the general population and do not specifically account for menopause-related variables or the nuances associated with HRT initiation.^19^ These tools may underestimate risk in younger postmenopausal women and do not incorporate menopause-specific variables such as age at menopause, time since menopause onset, vasomotor symptom severity, or HRT formulation and route. Furthermore, they do not consider reproductive history elements such as preeclampsia, gestational diabetes, or early surgical menopause that are increasingly recognized as important predictors of future CVD.^13,29^

**Figure 2:**
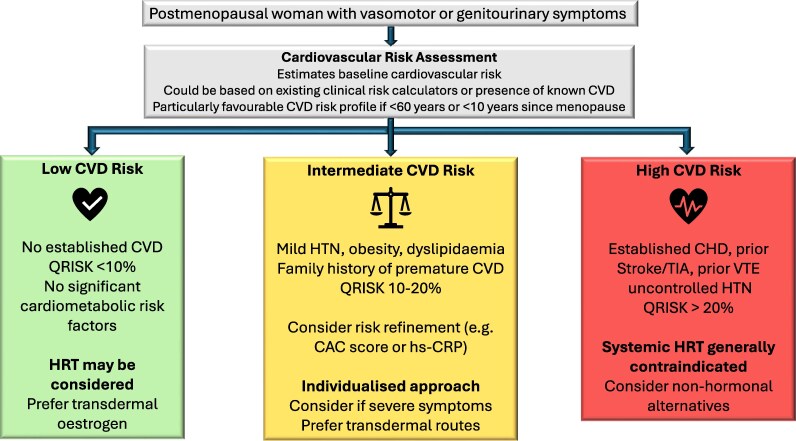
Risk stratification approach to HRT in postmenopausal women. Low-risk women may be considered for systemic HRT, ideally within 10 years of menopause, while high-risk women (established CVD or major risk factors) should generally avoid systemic HRT. Intermediate-risk women require individualized assessment and shared decision-making. Transdermal oestrogen and micronized progesterone are associated with more favourable CVD profiles than oral oestrogen or synthetic progestins. CAC, calcium artery calcification; CVD, cardiovascular disease; CHD, coronary heart disease; HRT, hormone replacement therapy; hs-CRP, high-sensitivity C reactive protein; HTN, hypertension; TIA, transient ischaemic attack; QRISK, QRISK cardiovascular risk score.

Development of a menopause-specific cardiovascular risk assessment tool may help address these limitations by integrating both traditional risk factors (e.g. age, blood pressure, lipid profile, diabetes, smoking status, and family history of CVD) and menopause-specific variables. These could include whether menopause occurred naturally or surgically, the number of years since menopause began, the presence and severity of menopausal vasomotor symptoms, and the intended HRT regimen (e.g. oral vs. transdermal oestrogen, type of progestogen, and treatment duration). Optional enhancements, such as coronary artery calcium (CAC) scoring or inflammatory markers like high-sensitivity C-reactive protein (hs-CRP), could further refine risk assessment, especially in borderline cases.^14,17,22^ A basic proposed decision framework is provided in *Figure 2*.

The development and implementation of such a tool could have significant clinical utility. It would enable personalized, evidence-based decision-making by stratifying women into low, intermediate, or high cardiovascular risk categories. For example, women identified as low risk could proceed with HRT using any standard regimen, while those in the intermediate category might benefit from transdermal oestradiol and micronized progesterone, which are associated with a more favourable cardiovascular and thrombotic risk profile. In contrast, women at high risk could be guided towards non-hormonal alternatives for symptom management. In addition to improving patient safety, a menopause-specific calculator would support shared decision-making and increase clinician confidence. Overall, such a tool would represent a significant advancement in precision medicine for midlife women’s health.

## Conclusion

Hormone replacement therapy has nuanced cardiovascular effects that depend heavily on timing of initiation, hormone type, administration route, and individual risk factors. Evidence from key trials suggests that starting HRT within 10 years of menopause or before age 60 does not appear to increase cardiovascular risk in most healthy women and may offer cardiovascular benefit and reduce mortality risk, whereas later initiation may increase risk. Current guidelines emphasize individualized treatment plans based on a comprehensive evaluation of benefits, risks, and patient preferences. However, important knowledge gaps remain, particularly regarding the long-term cardiovascular safety of newer hormone formulations and their effects in diverse populations.
